# A Cow with Chronic Cough That Did Not React

**Published:** 1896-04

**Authors:** Louis Olry Lusson

**Affiliations:** Ardmore, Pa.


### A COW WITH CHRONIC COUGH THAT DID NOT REACT AFTER REPEATED INJECTIONS OF TUBERCULIN.

BY LOUIS OLRY LUSSON, V.M.D.,
ARDMORE, PA.

THE mother of the cow above mentioned, some six years ago, developed a persistent bronchial cough that would not respond to treatment. She was then carrying the heifer (her first calf). I became alarmed at this condition of the mother and told the owner of my suspicions, but, as she was milking well, delayed the execution for four years—at which time she was tested with tuberculin and gave a reaction of $2\frac{2}{5}$° F. The daughter about this time also developed a cough similar to the mother. She was also tested at this time (about two years ago), but only reacted about $\frac{2}{5}°$ F., her normal temperature being about 102° F. My client decided to kill the mother but *not* the daughter. The autopsy revealed right lung entirely hepatized over mass of cheesy degeneration, with cavities here and there containing pus, air entirely occluded; left lung, anterior lobe one large cavity containing about one quart of pus; medium lobe, tubercles and small abscess, entirely hepatized; posterior lobe containing some tubercles in the first stages. The pleura and entire thoracic cavity literally covered with tubercles, with some on the omentum. If I remember correctly, the liver contained two or three large tubercles. As will be seen from result of autopsy the animal was doing all her oxygenating through the posterior lobe of left lung, but was in fair condition and milking some six or eight quarts daily.

After result of autopsy above noted, and considering that the daughter had been for six years the constant companion of the mother, continually breathing the same atmosphere, I concluded that she also must be tuberculous, but the owner, a physician of prominence, stood loyally by tuberculin and refused to have her killed. About the middle of February of this year I was again requested to test her, and before and after injection the temperature registered 102° F. Fifteen hours after the introduction of tuberculin the temperature had dropped to 101° F., and at twenty-one hours had gotten back to 102° F. Ten days later I made another injection of a new invoice of tuberculin, and this time the temperature was the same, 102° F.; before six hours later had dropped $\frac{3}{5}°$ F., and fell until at the twentieth hour registered 2° below temperature taken before injection. At the twenty-sixth hour temperature had gotten back to $101\frac{2}{5}°$ F. A few days later I destroyed the suspect. Autopsy did not reveal the slightest sign of tuberculosis in any of the organs, but the large and small bronchi in both lungs contained finely masticated food, in some cases packed in the bronchi very tightly, dry, and hard, occluding the air; in other, and most particularly the larger, tubes, looser and mixed with pus, and every evidence of chronic bronchitis, ever irritated by this foreign substance.

From the facts noted I am impressed with the idea that tuberculin has an antifebrile action in cases where there is febrile condition without the presence of tuberculosis, and, further, a sedative action upon the lungs; as this subject was always easier for several days after the injection, and, still further, that the introduction of tuberculin into the bovine species is perfectly harmless unless tuberculosis is already present, even though the subject was carried by a tuberculous mother; and, in conclusion, that tuberculin is an infallible test.

